# Applied Physiology for Advanced Grades

**Published:** 1898-03

**Authors:** 


APPLIED PHYSIOLOGY FOR ADVANCED GRADES—Including the Effects of Alcohol and Narcotics. By Frank Overton, A. M., M. D., Late House Surgeon to the City Hospital, New York. Cloth. 12mo, 432 pages. With Illustrations and Diagrams. Price, 80 cents. American Book Company, New York, Cincinnati and Chicago.

This book has been prepared to meet the requirement of teachers and schools for a modern text-book of Applied Physiology, which should embody the latest results of study and research in biological and chemical science, and the best pedagogical methods in science teaching. It represents a new and radical departure from the old-time methods pursued in teaching physiology. It begins with the study of the cells of the body as the units in which life exists and acts, and with this as the basis of treatment, shows their relation to all the elements of the human body and all the processes of human action. This is the keynote of the treatment throughout the book, and is the first attempt to apply, in a consistent and scientific manner, the principles and facts of biology to the study and teaching of physiology in schools.

Other distinctive and valuable features of the book are the arrangement of summaries and review topics at the end of each chapter; the list of subjects for original demonstrations and the use of the miscroscope in connection with the study; the special treatment of hygienic subjects, as air, ventilation, drinking water, sewage, clothing, bathing, bacteria, repair of injuries, etc. The effects of alcohol and narcotics are treated in a judicious and scientific manner, not in a separate chapter, but in connection with the several topics and subjects treated in the book. The book is not only modern and scientific in treatment, but it is written in such a clear and direct style as to make every subject interesting and comprehensible. The topical arrangement and clear typography of the pages will render the use of the book convenient and satisfactory.

---

The *American Monthly Review of Reviews* for March publishes three important interviews concerning the anti-Jewish crusade in France. The first, with M. Drumont, the head and front of the anti-Semetic agitation in France, is reported by Valerian Gribayedoff, the well-known Russian journalist and artist, formerly of New York. Robert H. Sherard reports conversations with Dr. Max Nordau, the author of "Degeneration," and with M. Zola; these, of course, give the Jews' side of the story. The whole series, taken together, throws much light on what to the Anglo-Saxon mind seems so incomprehensible—the real animus of French anti-Semitism, and especially its bearing on the Dreyfus case and Zola's trial in Paris.

